# Cure of Cancer

**Published:** 1896-09

**Authors:** 


Cure of Cancer. Six months ago important news came from Paris to the medical world in regard to the new method of treating cancer by serum-therapy. Drs. Richet and Hérricourt have, according to their reports before the Academy of Sciences in Paris, succeeded in discovering this medicine. The serum is obtained by injection of the cancer-poison from mules, and experiments have been made which gave astonishingly favorable results. Lately Drs. Richet and Hérricourt have continued their experiments on fifty patients. From these observations it follows that the pain is often quickly and permanently relieved by injections of serum; the growth becomes decidedly reduced in size; the development of the disease is much more slow, and finally the general condition is so much improved that patients who had already been given up were able to live three or four months longer in comparative comfort. The injections are harmless in themselves—*i. e.*, they produce no local disturbance except the slight eruptions which all serums do. The two investigators draw the conclusion that the cancer-serum is not able to radically cure malignant new-formations, but it influences them in a more favorable way than any known medicine.—*Thierärzt. Centralblatt*, March 1, 1896.

